# Peripheral nodulocystic corneal degeneration: a case report

**DOI:** 10.1016/j.ajoc.2026.102617

**Published:** 2026-06-11

**Authors:** Mariana Arino, Maria Lee, Jillian Chong, Madeline Yung

**Affiliations:** a Department of Ophthalmology, University of California, San Francisco, CA, USA; b Beverly Hills Institute of Ophthalmology, USA

**Keywords:** Peripheral nodulocystic corneal degeneration, Corneal nodules, Corneal degenerative disorders, Corneal edema

## Abstract

**Purpose:**

Peripheral nodulocystic corneal degeneration (PNCD) is a rare and poorly understood corneal degeneration. We report a case most consistent with PNCD in a patient with a remote history of conventional laser-assisted in situ keratomileusis (LASIK) and multiple systemic inflammatory conditions.

**Observation:**

A 52-year-old woman with a medical history of multiple systemic arthralgias and an ocular history of bilateral LASIK presented with progressive left eye dryness and discomfort. Ocular examination revealed multiple bullous corneal nodules in both eyes, predominantly in the superior and temporal periphery of the cornea. Anterior segment optical coherence tomography (AS-OCT) revealed subepithelial cystoid spaces with Bowman's Membrane disruption, and specular microscopy showed mild endothelial cell loss with polymegethism and pleomorphism. The clinical findings were most consistent with peripheral nodulocystic degeneration (PNCD). She declined superficial keratectomy, and her symptoms improved with topical cyclosporine, perfluorohexyloctane, and brimonidine.

**Conclusion and importance:**

We describe a case of lucent peripheral corneal bullae most consistent with PNCD, of which only one similar case has been previously reported in the literature. PNCD appears to be an indolent, slowly progressive corneal degeneration. This case highlights a unique presentation of corneal degeneration and cause of peripheral corneal bullae. The interplay between corneal degeneration, systemic inflammatory disease, and prior refractive surgery warrants further investigation.

## 1 Introduction

Peripheral nodulocystic corneal degeneration (PNCD) is an extremely rare corneal condition characterized by bilateral lucent nodules and cysts localized to the peripheral cornea. Its clinical course, risk factors, and effective treatments remain poorly defined. To date, only one case describing a similar presentation of PNCD exists in the literature.^1^

We report the clinical findings of an adult female with an acquired, bilateral corneal degeneration consisting of medium-large lucent bullae located in the peripheral cornea, most consistent with PNCD.

## 2 Case

A 52-year-old woman presented with bilateral ocular dryness and discomfort. Her past medical history was significant for polyarthralgia, fibromyalgia, and cervical and lumbar spondylosis. She was evaluated by rheumatology with a negative HLA-27 and ANA serology, with a final diagnosis of fibromyalgia. She reported a family ocular history of glaucoma, but no family history of corneal dystrophy or other corneal degeneration. Her past ocular history was notable for bilateral conventional LASIK with microkeratome and subsequent mild regression, but did not require corrective eyewear.

**Fig. 1:**
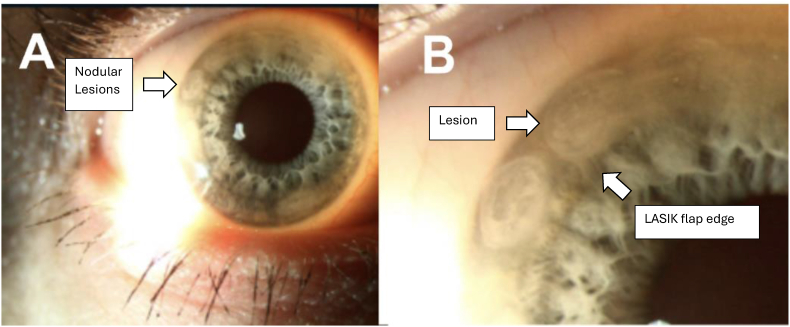
(A) Slit Lamp Photos of the right eye showing lucent bullae in the peripheral cornea (B) On higher magnification, the bullae, located peripheral to the LASIK flap edge, appear to have cystic spaces with normal underlying stroma and no associated inflammation.

**Fig. 2:**
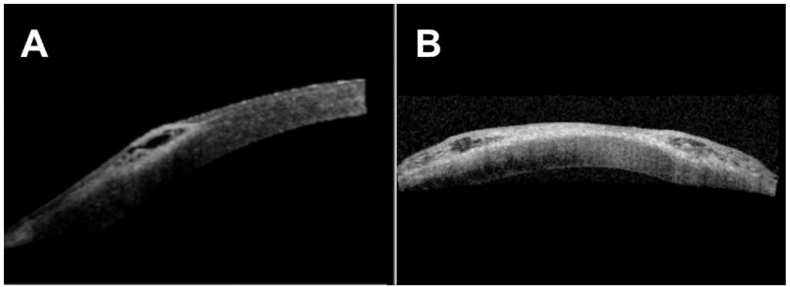
(A) Representative AS-OCT cross-sections of the right eye and (B) left eye demonstrate multiple hyperreflective corneal lesions consisting of corneal cysts with mild anterior stromal fibrosis. The underlying posterior corneal stroma appears normal without significant edema.

**Fig. 3:**
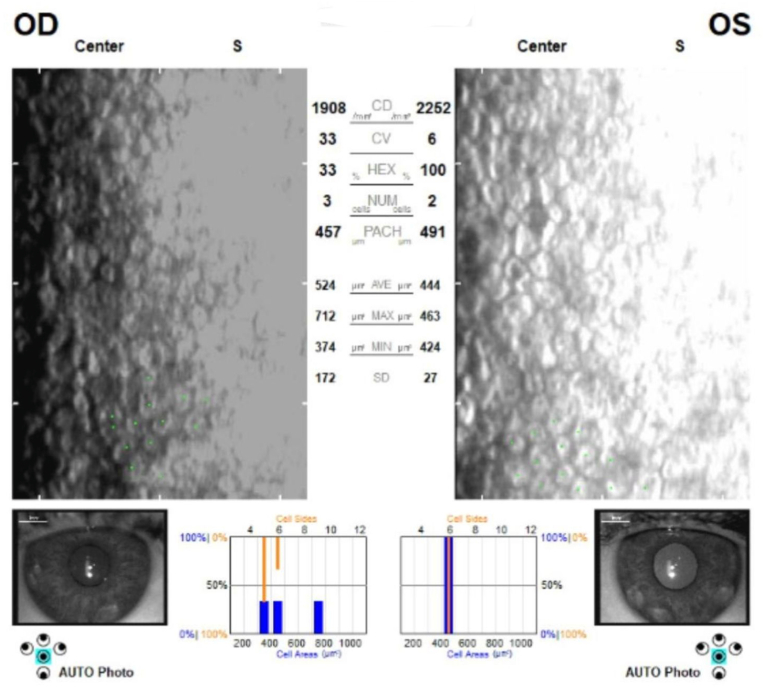
Specular Microscopy of right and left eye demonstrating mild bilateral corneal endothelial cell loss with associated polymegethism and pleomorphism.

On examination, the patient's uncorrected visual acuity was 20/25 in the right eye and 20/30, improving to 20/25 with pinhole in the left eye. Slit lamp examination of both eyes revealed peripheral corneal cystic nodules primarily outside of the LASIK flap edge, worst in the superior and temporal regions, measuring up to 2mm in width (Fig. 1A). There was a clear zone between the lesions and the limbus. These nodules appeared translucent, similar to bullae (Fig. 1), and remained stable without progression over the two-year follow-up period. Additional findings included mild tear film insufficiency, pigmented guttae, corneal scar, and Central Cloudy Dystrophy of Francois to both eyes. The central corneal thickness was 480 μm in the right eye and 491μm in the left eye. Anterior segment optical coherence tomography (AS-OCT) images revealed hyperreflective subepithelial cystoid regions extending into the anterior stroma (Fig. 2). Alterations to Bowman's Membrane were additionally observed in imaging. Specular microscopy demonstrated mild bilateral corneal endothelial cell loss with associated polymegethism and pleomorphism, more pronounced in the right eye, consistent with early endothelial dysfunction (Fig. 3). All other slit lamp examinations of the conjunctiva, sclera, anterior chamber, iris, lens, and anterior vitreous were normal.

The patient was trialed on cyclosporine, perfluorohexyloctane, and brimonidine with partial symptomatic improvement. A superficial keratectomy was offered to the patient. She declined the procedure and preferred to continue topical medications.

## 3 Discussion

In this case, a patient presented with atypical peripheral cystic corneal nodules that resist clear classification within established degenerative or post-surgical diagnoses. To the author's knowledge, only one case with comparable features has been previously reported in the literature.

This presentation is strikingly similar to a case of circumferential nodulocystic keratopathy reported in 1988, as demonstrated by lucent, peripheral bullae that spare the visual axis without significant stromal opacity or vascularization (Table 1).^1^ The nodules were arranged in an arcuate pattern in both the superior and inferior cornea, with a clear zone between the lesions and the limbus. The nodules were notably large (2–4 mm), exhibited cystic characteristics, and were occasionally confluent. The patient underwent superficial keratectomy without recurrence. On histology, the corneal epithelium remained intact aside from focal spongiotic intercellular edema, and there was no evidence of intraepithelial inflammation or necrosis. Subepithelial changes consisted of serous fluid collections, while the underlying stromal lamellae were unremarkable and Bowman's layer remained intact. The patient did have a history of regional ileitis, rheumatoid arthritis, and ankylosing spondylitis. There was no report of guttae or other histologic features consistent with endothelial compromise. The peripheral distribution, presence of a clear zone between the lesions and limbus, cystic morphology, association with inflammatory diseases, and lack of alternative explanatory findings in our patient are most consistent with the previous description of PNCD and likely represent the second case reported in the literature to our knowledge.

**Table 1:** Comparative clinical and structural features of the current case presented versus previously reported case.

Features	Current Case	1988 Case Report
Clinical Course	Bilateral ocular dryness and discomfort; stable over 2 years without surgical intervention	Bilateral presentation; treated with superficial keratectomy without recurrence
Visual axis involvement	Not involved	Not involved
Exam findings	Bilateral, peripheral cystic nodules (superior and temporal), clear zone between lesions and limbus, occasionally confluent, peripheral to LASIK flap edge	Bilateral peripheral cystic nodules (superior and inferior), clear zone between lesions and limbus, occasionally confluent, clear zone between lesions and limbus
Lesion size	2 mm	2–4 mm
AS-OCT	Hyperreflective subepithelial cystoid regions extending into anterior stroma, alterations of Bowman's membrane observed	Not available
Histopathology	Not performed	Intact epithelium with focal spongiotic edema, no intraepithelial inflammation or necrosis
Bowman's layer	Disrupted on AS OCT	Intact on histology
Anterior stroma	Subepithelial cystoid changes extending into anterior stroma	Subepithelial serous fluid collections, stroma otherwise remarkable
Endothelium	Mild bilateral endothelial cell loss with polymegethesim, pigmented guttae present	No reported guttae or endothelial compromise on histology
Systemic associations	Polyarthralgia, fibromyalgia, cervical/lumbar spondylosis, no defined autoimmune diagnosis	Regional ileitis, Rheumatoid arthritis, ankylosing spondylitis

The differential diagnosis for peripheral corneal nodules includes Salzmann nodular degeneration and peripheral hypertrophic subepithelial corneal degeneration (PHSCD). Salzmann nodules are bluish-white to gray corneal elevations that often follow prior keratitis, autoimmune, or ocular surface disease, and they histologically feature epithelial thinning, disruption of Bowman's layer, and anterior stromal fibrosis.^1^ PHSCD is a bilateral, symmetrical disease characterized by peripheral circumferential fibrotic subepithelial lesions that spare the central cornea and show no inflammation on histopathology, primarily affecting middle-aged white women.^2^ Salzmann nodules and PHSCD have also been reported as a late complication of LASIK.^3^, ^4^, ^5^ However, Salzmann nodules and PHSCD do not have a bullous morphology and are histologically distinct from PNCD.^1^

Etiologies of corneal bullae due to endothelial dysfunction include Fuchs’ Endothelial Dystrophy (FECD) and Brown-McLean Syndrome (BMS). FECD is a non-inflammatory, sporadic or autosomal dominant dystrophy involving the corneal endothelium, causing guttae, epithelial bullae, and stromal edema.^6^ However, FECD classically presents with central, rather than peripheral, corneal edema. Brown-McLean Syndrome (BMS) is a well-described cause of peripheral corneal edema arising as a delayed complication of intraocular surgery, especially procedures involving large limbal or corneal incisions such as intracapsular cataract extraction.^7^ BMS often is associated with corneal guttae and iridodonesis, though endothelial cell count and morphology are preserved.^7^^,^^8^

In addition to peripheral corneal bullae, this patient did present with central guttae and mild endothelial dysfunction on specular microscopy. Although a causal relationship cannot be established, LASIK has been shown to decrease endothelial cell density, which may potentially contribute to fluid removal dysfunction and bullae formation, especially in the setting of underlying FECD.^9^^,^^10^ While guttae are commonly associated with edema, they have also been linked to stromal fibrosis.^11^ In our report, AS-OCT demonstrated hyperreflectivity of the corneal endothelium, whereas fibroblast proliferation was noted in the previously reported case. However, the previous case of PNCD was not associated with endothelial dysfunction, and neither case demonstrated stromal edema – it remains unclear whether endothelial dysfunction is the underlying mechanism for PNCD.

Outside of endothelial disease, corneal cysts have been associated with congenital epithelial invasion during embryonic development, as well as acquired causes such as penetrating trauma or surgical disruption of the epithelium described.^12^^,^^13^ In the case reported in 1988, histopathology demonstrated that the nodules contained collagen bundles, albumin, and eosinophilic, homogeneous serous fluid pools. In our case, the nodule contents appeared lucent, but with similar characteristics, suggesting serous accumulation. The presence of proteinaceous edematous fluid in addition to mechanisms of fluid migration remains uncertain. The presence of albumin and other contents may generate osmotic pressure, thereby contributing to the formation of corneal cysts in both cases.

Nodule formation has also been associated with inflammatory processes, such as previous episodes of conjunctivitis, keratitis, or uveitis, as well as systemic inflammatory conditions like inflammatory bowel disease or arthritic disorders.^10^^,^^14^ In the previous case report, the patient was diagnosed with inflammatory bowel disease (regional ileitis) and rheumatoid arthritis with ankylosing spondylitis. The authors posited that systemic inflammation may contribute to the disruption of corneal integrity and progressive nodular lesions. Our patient presented with polyarthralgia, fibromyalgia, cervical and lumbar spondylosis, without clear autoimmune etiology on rheumatologic evaluation. While these findings raise the possibility of inflammatory drivers of nodule formation, though the relation of PNCD to systemic inflammation remains unclear and should therefore be interpreted cautiously.

The patient elected conservative management after declining superficial keratectomy, which had been successfully performed in the previously reported case. Accordingly, medical management was pursued, including brimonidine for reduction of erythema, and cyclosporine and perfluorohexyloctane to mitigate ocular surface irritation from the elevated nodules. The regimen appeared to provide symptomatic relief from discomfort associated with the nodular lesions, but did not result in the regression of the corneal lesions. Therefore, conservative management with lubricants may be sufficient for mild cases without visual impairment, while surgical intervention may be warranted when nodular lesions encroach on the visual axis and reduce visual acuity.^15^ Superficial curettage of the nodules could be a plausible first step should surgical intervention become necessary, avoiding the need for more invasive procedures such as lamellar or penetrating keratoplasty.

In conclusion, this case represents a rare and atypical presentation of cystic corneal nodules most consistent with PNCD or a closely related nodulocystic corneal degeneration, a manifestation described only once previously in the literature. Recognition of this entity is important to distinguish it from more common degenerations and postsurgical complications. Further study is warranted to clarify its pathogenesis, including the roles of endothelial integrity, stromal architecture, and systemic inflammation or surgical risk factors.

## CRediT authorship contribution statement

**Mariana Arino:** Writing – review & editing, Writing – original draft, Formal analysis, Conceptualization. **Maria Lee:** Writing – review & editing, Writing – original draft, Conceptualization. **Jillian Chong:** Writing – review & editing. **Madeline Yung:** Writing – review & editing, Writing – original draft, Supervision, Formal analysis, Conceptualization.

## Declarations and patient consent

The study adheres to the tenets of the Declaration of Helsinki. The patient provided written consent for the publication.

## Authorship

All authors attest that they meet the current ICMJE criteria for Authorship.

## Artificial intelligence statement

No artificial intelligence–assisted technologies were used in the generation, analysis, writing, or editing of this manuscript.

## Funding

No funding or grant support

## Declaration of competing interest

The authors declare that they have no known competing financial interests or personal relationships that could have appeared to influence the work reported in this paper.

## List of abbreviations

PNCD –: Peripheral Nodulocystic Corneal Degeneration
BSK: Band-shaped keratopathy
LASIK: laser-assisted in situ keratomileusis
BCVA: Best corrected visual acuity
OCT: Optical coherence tomography

## References

[1] Carpel E.F., Cameron J.D., Wick M.R. (1988). Circumferential nodulocystic keratopathy. A case report. Graefes Arch Clin Exp Ophthalmol.

[2] Moshirfar M., Moin K.A., Ronquillo Y. (2024 Oct 6). Statpearls [Internet].

[3] Stem M.S., Hood C.T. (2015). Salzmann nodular degeneration associated with epithelial ingrowth after LASIK treated with superficial keratectomy. BMJ Case Rep.

[4] Barsam C.A., Olson R.J., Moshirfar M. (2004). Bilateral Salzmann's Nodular–like degeneration occurring following LASIK. Two case reports. Investig Ophthalmol Vis Sci.

[5] Hopping G.C., Somani A.N., Vaidyanathan U. (2020). Myopic regression and recurrent Salzmann nodule degeneration after laser in situ keratomileusis in Ehlers-Danlos syndrome. Am J Ophthalmol Case Rep.

[6] Ayres B.D., Feldman B.H., Patel A.S. (2025). Fuchs' endothelial dystrophy. EyeWiki Am Acad Ophthalmol.

[7] Kam K.W., Jhanji V., Young A.L. (2013). Brown-McLean syndrome. BMJ Case Rep.

[8] Gothard T.W., Hardten D.R., Lane S.S., Doughman D.J., Krachmer J.H., Holland E.J. (1993). Clinical findings in Brown-McLean syndrome. Am J Ophthalmol.

[9] Ljubimov A.V., Saghizadeh M. (2013). Progress in corneal wound healing. Prog Retin Eye Res.

[10] Jones S.S., Azar R.G., Cristol S.M. (1998). Effects of laser in situ keratomileusis (LASIK) on the corneal endothelium. Am J Ophthalmol.

[11] Gurnani B., Somani A.N., Moshirfar M., Patel B.C. (2025 Apr 6). Statpearls [Internet].

[12] Chen A.C., Nowroozizadeh S., Kedhar S., Minckler D. (2020). Trans-stromal epithelial cyst after multiple lamellar keratoplasties – case report and review of literature. Am J Ophthalmol Case Rep.

[13] Kim S.W., Kim E.K. (2017). Portable OCT-assisted surgical treatment of intracorneal pre-descemet epithelial cyst: a case report. BMC Ophthalmol.

[14] Sirajeldin A., Hwang F.S., Bunya V.Y. (2025 Feb 11). https://eyewiki.org/Salzmann_Nodular_Degeneration.

[15] Moshirfar M., Moin K.A., Ronquillo Y. (2025). Statpearls.

